# Supplementary material for: DockingApp RF: A State-of-the-Art Novel Scoring Function for Molecular Docking in a User-Friendly Interface to AutoDock Vina
Source: Int J Mol Sci. 2020 Dec 15;21(24):9548. doi: 10.3390/ijms21249548 (PMC7765429; doi:10.3390/ijms21249548)
Supplement: Supplementary file 1 [file ijms-21-09548-s001.pdf]

International Journal of *Molecular Sciences*

MDPI

Article

DockingApp RF: A State-of-the-Art Novel Scoring Function for Molecular Docking in a User-Friendly Interface to AutoDock Vina

Gabriele Macari [1], Daniele Toti [2], Andrea Pasquadibisceglie [1] and Fabio Polticelli [1,3,*]

[1] Department of Sciences, Roma Tre University, 00146 Rome, Italy; gabriele.macari@uniroma3.it (G.M.); andrea.pasquadibisceglie@uniroma3.it (A.P.)

[2] Faculty of Mathematical, Physical and Natural Sciences, Catholic University of the Sacred Heart, 25121 Brescia, Italy; daniele.toti@unicatt.it

[3] National Institute of Nuclear Physics, Roma Tre Section, 00146 Rome, Italy

* Correspondence: fabio.polticelli@uniroma3.it

Received: 26 November 2020; Accepted: 11 December 2020; Published: date

1. Supplementary Methods

1.1. Energy terms

The energy terms used for Docking App RF's features selection can be found in the following 3 Eq. 1, Eq. 2, Eq. 3 and Eq. 4. These equations are taken from [1] and calculated by using the option 4 score_only of AutoDock Vina.

Gaussian terms:

$$\begin{aligned} Gauss_1 &= e^{-((d-o_1)/s_1)^2} \\ Gauss_1 &= e^{-((d-o_2)/s_2)^2} \end{aligned} \quad (1)$$

Repulsion term:

$$Repulsion(d) = \begin{cases} d^2, & for\ d \le 0 \\ 0, & for\ d > 0 \end{cases} \quad (2)$$

Hydrogen bond term:

$$HBond(d) = \begin{cases} 1, & for\ d \le h_1 \\ \dfrac{d}{-h_{1,}}, & for\ h_1 < d < 0 \\ 0, & for\ d \ge 0 \end{cases} \quad (3)$$

Hydrogen bond term:

$$Hydrophobic(d) = \begin{cases} 1, & for\ d \le p_1 \\ p_2 - d, & for\ p_1 < d < p_2 \\ 0, & for\ d \ge p_2 \end{cases} \quad (4)$$

1. Trott, O.; Olson, A.J. AutoDock Vina: improving the speed and accuracy of docking with a new scoring function, efficient optimization, and multithreading. J Comput Chem **2010**, 31, 455–61.

1.2. Ten-fold Cross-validation

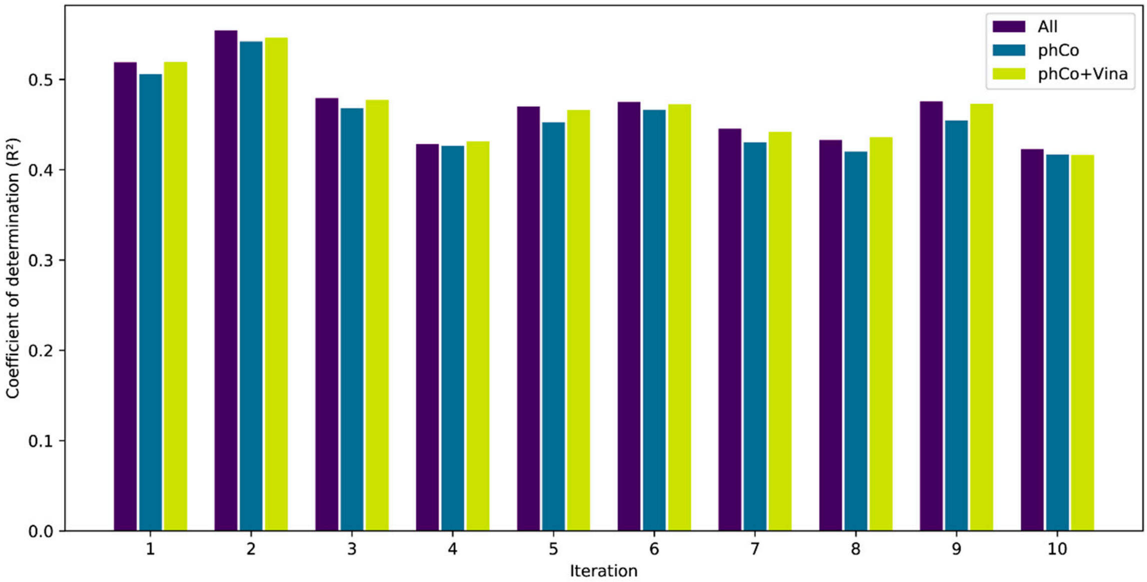

Figure 1. Results of the ten-fold cross-validation performed for three models trained with three different combinations of features: only intermolecular contacts (phCo, light blue), phCo plus Vina energy terms (green), phCo with Vina and solvent accessible surface area feature (dark blue, All).

1.3. Docking power

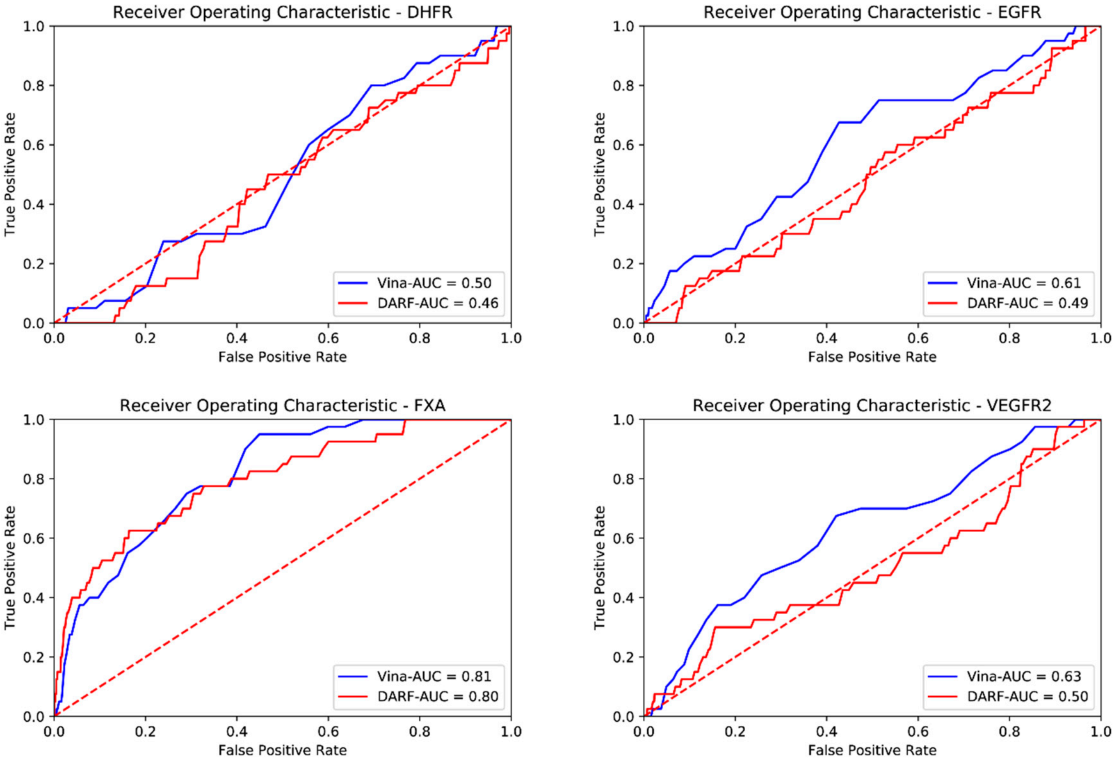

Figure 2. ROC curve and AUC score for the four challenging complexes derived from DEKOIS2.0. The ROC curve for DockingApp RF (DARF) is depicted in red, while the one for Vina in blue.

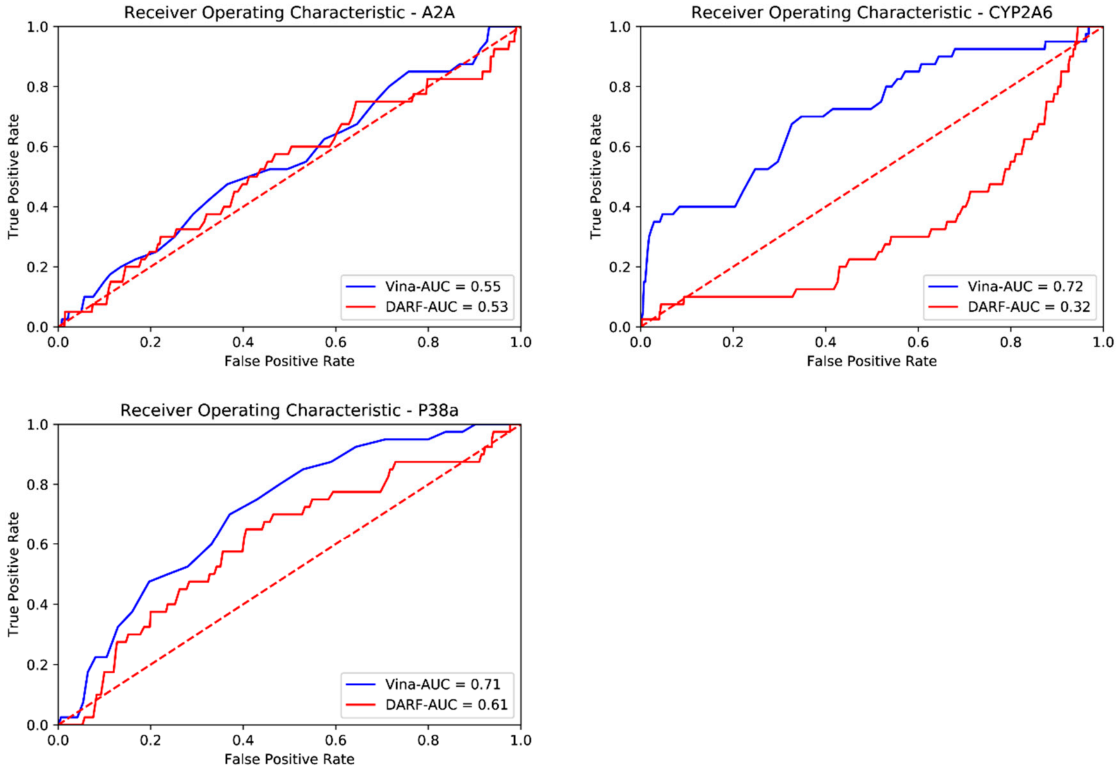

Figure 3. ROC curve and AUC score for the three moderately challenging complexes derived from DEKOIS2.0. The ROC curve for DockingApp RF (DARF) is depicted in red, while the one for Vina in blue.

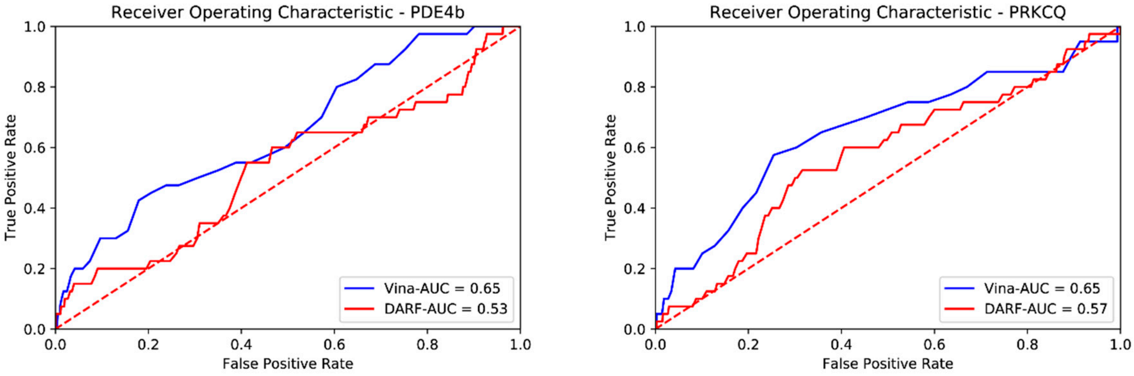

Figure 4. ROC curve and AUC score for the two less challenging complexes derived from DEKOIS2.0. The ROC curve for DockingApp RF (DARF) is depicted in red, while the one for Vina in blue.

1.4. Features importance

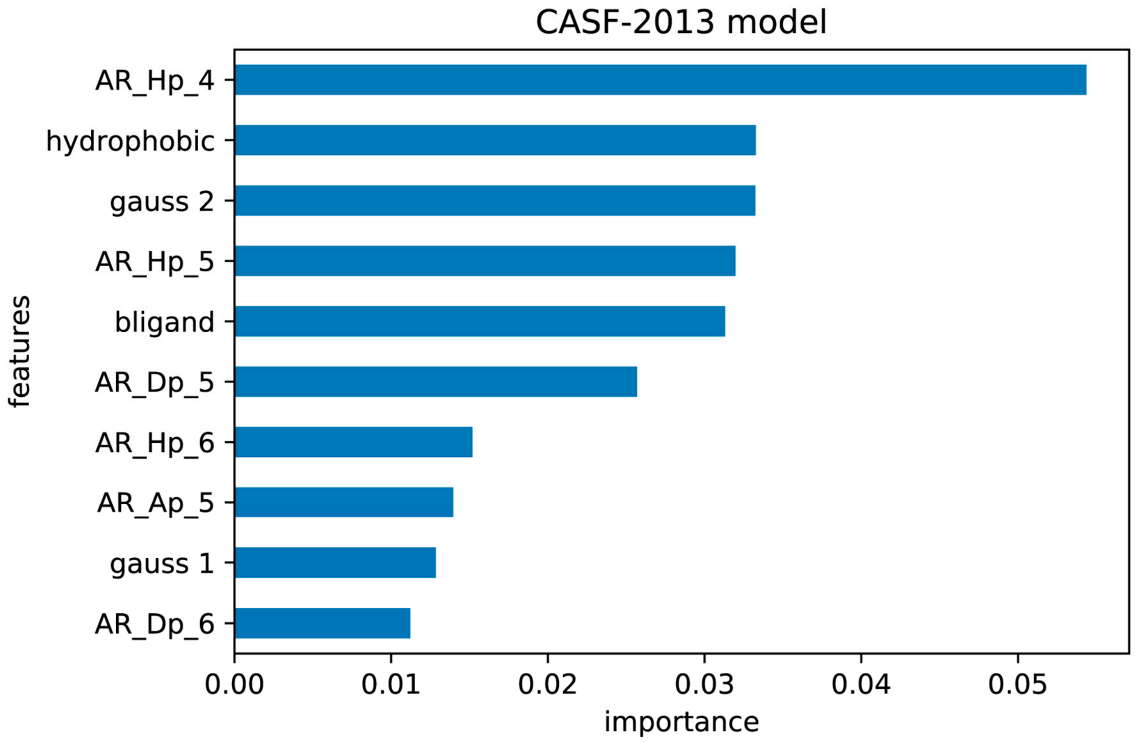

Figure 5. The ten most important features in the CASF-2013 model. PhCo features are presented with the format X_Yp_n, where X is the pharmacophoric feature calculated on a ligand atom, Y the pharmacophoric feature calculated on a protein atom, hence the p, and n is the shell at which the contact is registered.

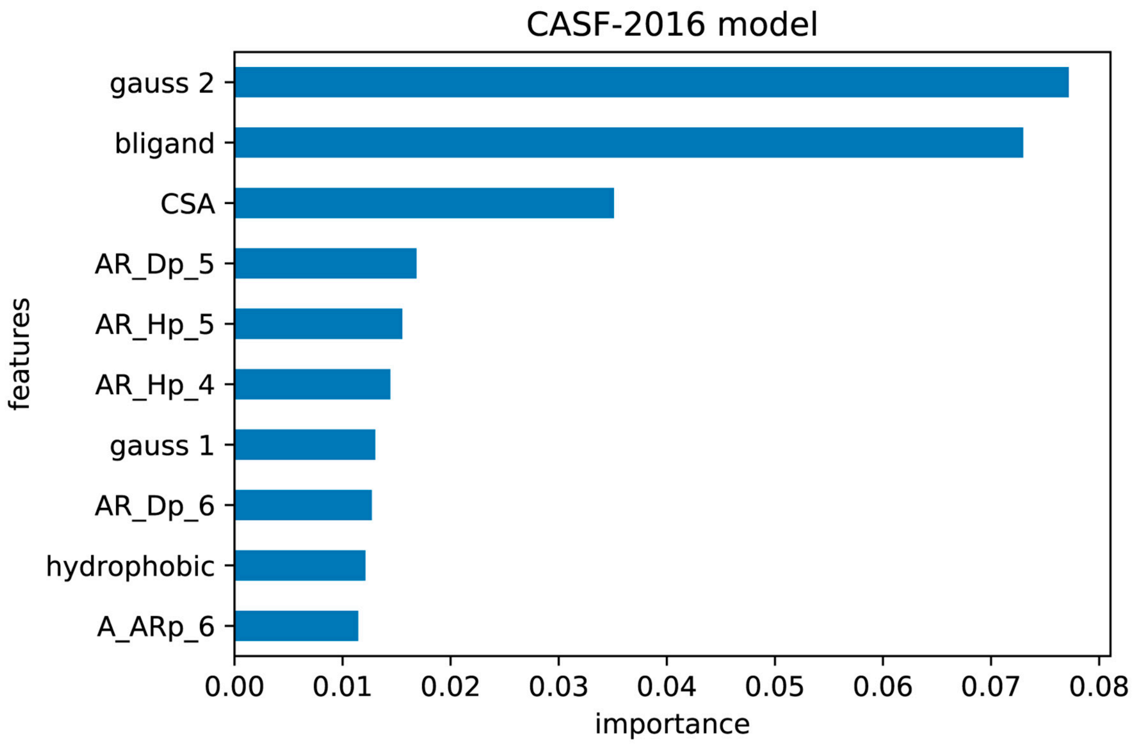

Figure 6. The ten most important features in the CASF-2016 model. PhCo features are presented with the format X_Yp_n, where X is the pharmacophoric feature calculated on a ligand atom, Y the pharmacophoric feature calculated on a protein atom, hence the p, and n is the shell at which the contact is registered.

1.5. Docking power

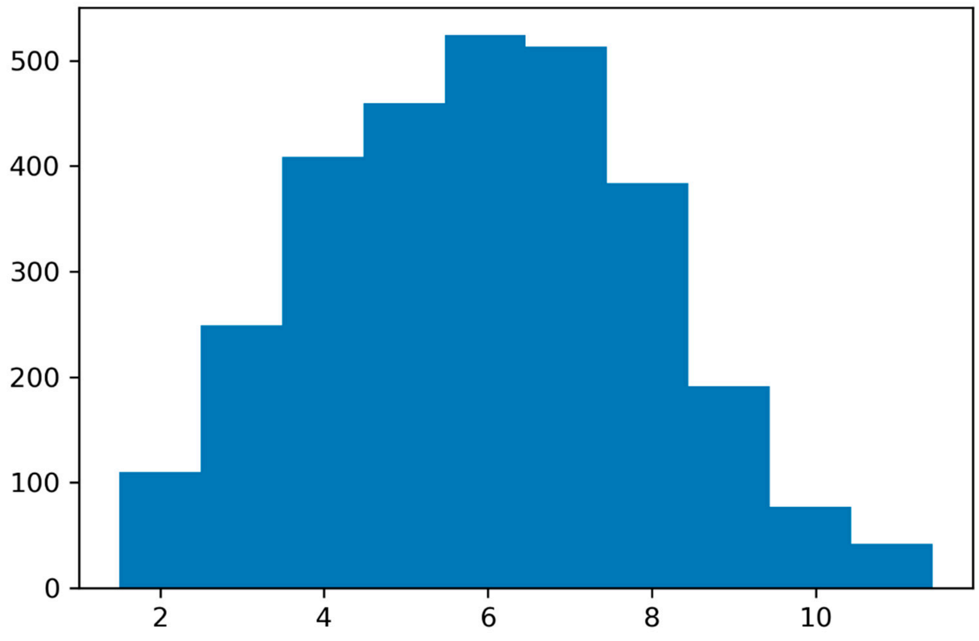

Figure 7. Distribution of the pKd values in the PDBBind2013 refined set.

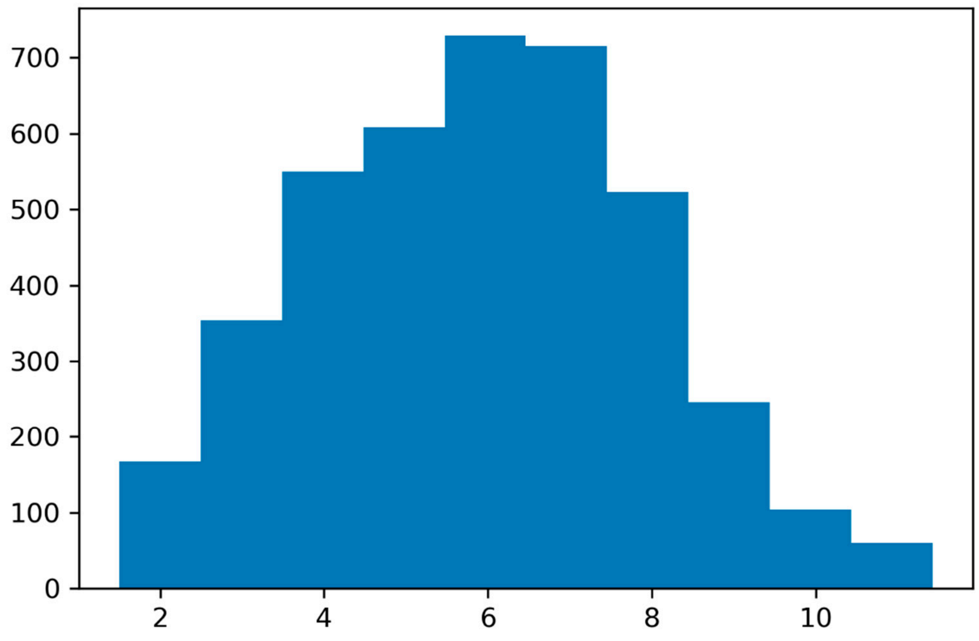

Figure 8. Distribution of the pKd values in the PDBBind2016 refined set.

© 2020 by the authors. Submitted for possible open access publication under the terms and conditions of the Creative Commons Attribution (CC BY) license (http://creativecommons.org/licenses/by/4.0/).
